# Food Insecurity with Hunger Is Associated with Obesity among HIV-Infected and at Risk Women in Bronx, NY

**DOI:** 10.1371/journal.pone.0105957

**Published:** 2014-08-27

**Authors:** Nicole Sirotin, Donald R. Hoover, Qiuhu Shi, Kathryn Anastos, Sheri D. Weiser

**Affiliations:** 1 Department of Medicine, Weill Cornell Medical College, New York, New York, United States of America; 2 Department of Statistics and Biostatistics, Institute for Health, Health Care Policy and Aging Research, Rutgers University, New Brunswick, New Jersey, United States of America; 3 Department of Epidemiology and Community Health, New York Medical College, Valhalla, New York, United States of America; 4 Departments of Medicine and Epidemiology and Population Health, Montefiore Medical Center and Albert Einstein College of Medicine, Bronx, New York, United States of America; 5 Division of HIV/AIDS, San Francisco General Hospital, University of California, San Francisco (UCSF), San Francisco, California, United States of America; Sookmyung Women’s University, Republic of Korea

## Abstract

**Background:**

Food insecurity, insufficient quality and quantity of nutritionally adequate food, affects millions of people in the United States (US) yearly, with over 18 million Americans reporting hunger. Food insecurity is associated with obesity in the general population. Due to the increasing prevalence of obesity and risk factors for cardiovascular disease among HIV-infected women, we sought to determine the relationship between food insecurity and obesity in this cohort of urban, HIV-infected and –uninfected but at risk women.

**Methods:**

Using a cross-sectional design, we collected data on food insecurity, body mass index and demographic and clinical data from 231 HIV-infected and 119 HIV-negative women enrolled in Bronx site of the Women’s Interagency HIV Study (WIHS). We used multivariate logistic regression to identify factors associated with obesity.

**Results:**

Food insecurity was highly prevalent, with almost one third of women (110/350, 31%) reporting food insecurity over the previous six months and over 13% of women reported food insecurity with hunger. Over half the women were obese with a Body Mass Index (BMI) of ≥30. In multivariate analyses, women who were food insecure with hunger had higher odds of obesity (Adjusted odds ratio [aOR] = 2.56, 95% Confidence Interval [CI] = 1.27, 5.20) after adjusting for HIV status, age, race, household status, income, drug and alcohol use.

**Conclusion:**

Food insecurity with hunger was associated with obesity in this population of HIV-infected and –uninfected, urban women. Both food insecurity and obesity are independent markers for increased mortality; further research is needed to understand this relationship and their role in adverse health outcomes.

## Introduction

Food insecurity, defined as uncertain availability of nutritionally adequate, safe food, affected 14.5% of American households in 2012. Almost 6% of American households were in the most severe category of food insecurity called very low food security or food insecurity with hunger [1]. Food insecurity with hunger exists when meals are skipped or decreased in size due to lack of resources to purchase adequate quantity or quality of food [1]. While in the developing world food insecurity is associated with being underweight [2], in developed countries, multiple cross-sectional studies have found a relationship between food insecurity and obesity, particularly among women [3]–[8].

The relationship between food insecurity and body mass index (BMI) is complex and varies by population and study [6]–[8]. For those who are often food insecure, during periods of adequate food availability there may be overconsumption of high calorie, energy dense foods, which can result in metabolic changes, such as insulin resistance, that predispose individuals to obesity [9], [10]. In a study in the general population in California, obesity was more prevalent among women who experienced food insecurity with hunger as compared to food secure women (35.2% vs. 16.2%, P = <0.05), particularly among minority women. Among black and Hispanic women who were food insecure with hunger, 52.1% and 42.1% respectively were obese, compared to 26.5% of white women [3]. Additionally, among the over 4000 women enrolled in the National Health and Nutrition Examination Survey (NHANES), food insecurity was associated with being overweight and food insecurity with hunger was associated with being obese [11]. Further studies have shown that in low income populations, food insecurity is associated with obesity-related cardiovascular risk factors such as self-reported hypertension (Adjusted Relative Risk (ARR) 1.20, 95% Confidence Interval (CI) [1.04–1.38]) [12], dyslipidemia (ARR 1.30, 95% CI [1.09–1.55]) and a higher odds of diabetes (Adjusted Odds Ratio (AOR) 2.1, 95% CI [1.1–4.0]) [12], [13]. Food insecurity is also associated with a 20% higher odds of inflammation as measured by elevated C Reactive Protein, an inflammatory marker, and through elevated white blood cells [14].

Due to known increases in cardiovascular risk factors, inflammation and immune activation among people with HIV, understanding the potential upstream causes of these risk factors is particularly important. Food insecurity is prevalent among US urban, HIV-infected, marginalized populations, with estimations that up to 50% of some of these HIV-infected populations experience food insecurity [15]–[17]. Among HIV-infected individuals, food insecurity is an important predictor of poor HIV treatment outcomes, morbidity and increased mortality [18], although the pathways that explain this relationship are still unclear [19]. Obesity, increasingly recognized as causing a high inflammatory state [20], is also an important cause of morbidity and mortality among women with HIV [21], [22], particularly among poor, minority women [3], [23].

Since obesity may further compound consequences of HIV on inflammation and cardiovascular risk factors, understanding the association between food insecurity and obesity is particularly important among HIV-infected populations. We therefore sought to determine the prevalence of food insecurity, and its relationship with obesity in urban, HIV-infected and at risk women enrolled in the Bronx site of Women’s Interagency HIV Study.

## Methods

### Ethics Statement

Each WIHS participant gave written informed consent and the institutional review board at Montefiore Medical Center approved of this study.

### Population

The Women’s Interagency HIV Study (WIHS) is a, multicenter cohort designed to study the effect of HIV infection on women [24]. In 1994, HIV-infected and HIV-negative women were recruited by institutional and community organizations and since then have been followed every 6 months. Trained research assistants collected socio-demographic data at study entry including age, income, education level, employment, and behavioral health risk factors. At baseline and each subsequent visit, participants had a focused physical examination, provided blood specimens for CD4 lymphocyte and complete blood counts, and provided information on recent health status, health behaviors, and socioeconomic status.

### Setting

This sub-study is limited to the Bronx WIHS site only. Bronx, NY was recently noted as having among the lowest incomes among urban counties in the country [25]. Almost 50% of all households and 56% of households with children in the South Bronx are recipients of federal Supplemental Nutrition Assistance Program (SNAP, formerly called Food Stamps) suggesting considerable food insecurity, and it is estimated that only 2/3 of people who are eligible actually receive benefits [26]. This sub-study consists of 231 HIV infected and 119 HIV-negative women at Visit 34 between April 2011 and, October 2011.

### Measures

With the exception of demographic variables collected at baseline visits, all variables for this analysis were obtained from visit 34.

The United States Department of Agriculture (USDA) Food Security Survey Module, a validated tool to measure levels of food security for the US population, was administered by research assistants trained with the USDA recommended probes [27]. The USDA Food Security Survey Module differentially assesses food security status level for both households with children and households without children through 18 and 10 questions, respectively. The questions assess both quality and quantity of food eaten and anxiety around not having food for the household (with the exception of deliberately lowering consumption to reduce weight) over the last 6 months. Households of participants were classified as food secure, food insecure without hunger, or food insecure with hunger based on a standardized scoring algorithm as recommended by the USDA [27]. We then collapsed this into food insecure with hunger versus all other categories based on prior studies showing associations between food insecurity with hunger and important clinical outcomes [12]–[14], [18].

We adjusted for relevant demographic variables previously found to be associated with food insecurity [15], [18], including age (continuous), participant’s income (<$6000 per year vs. $6000–$12,000 per year vs. >$12,000 per year), self-identified race/ethnicity: Black including both Black Hispanic and Black non-Hispanic; White including both White Hispanic and White non-Hispanic; and Other), housing (stable = own apartment or lived with family vs. not stable), current employment (yes vs. no), marital status (married/long term partner vs. other), having health insurance (yes vs. no) and whether the household included members under age 18. We also controlled for behavioral variables which have been previously associated with food insecurity and obesity [28] including: sexual risk behaviors during the past 6 months (having any unprotected sex acts versus none), drug (any cocaine, heroin and crack usage versus none) and alcohol use (none, low (<3 drinks per week), moderate (3–13 drinks per week), high (>14 drinks per week)).

Obesity, the primary outcome, was measured with Body Mass Index (BMI) calculated as weight divided by height-squared (kg/m^2^). BMI was dichotomized to <30 (not obese), ≥30 (obese) for the analysis.

### Statistical analysis

Statistical analysis was performed using SAS (version 9.2, SAS Institute Inc., Cary, NC, USA). We first compared sociodemographic and clinical variables by food insecurity status. Next, we used univariate and multivariate logistic regression to identify factors associated with obesity. We built multivariate logistic regression models using backward selection; variables with a p-value of ≤0.20 were removed from the model. We performed the Hosmer-Lemeshow goodness of fit test on the final multivariable model.

## Results

Of 350 women enrolled in the study, approximately two thirds of the sample reported incomes of <$12,000 (222/350) and 61% of participants identified as black and 23% as “Other” when asked their race (Table 1). About two thirds of woman were HIV-infected (231/350) while ∼40% had children <18 years old in their household. All participants had health insurance. The majority of women were stably housed (89.7%) and reported very little drug use (4.6%); 16.9% reported moderate alcohol use in the prior 6 months. Approximately one third of women reported unprotected sex in the prior 6 months (33.6%) with HIV negative women reporting twice as much unprotected sex as compared to HIV-infected (52.0% vs. 24.2%, p<0.0001).

**Table 1:** Characteristics of population with Food Insecurity with Hunger vs. No Hunger.

Parameter		Total	Food Insecure with Hunger	No Hunger*	p-value
		N = 350	N = 47 (13.4%)	N = 303 (86.6%)	
Age, mean		48.9	49.1	48.8	0.88
Race/Ethnicity	White**	56 (16.0%)	9 (16.1%)	47 (83.9%)	0.04
	Black***	212 (60.6%)	21 (9.9%)	191 (90.1%)	0.20
	Other	82 (23.4%)	17 (20.7%)	65 (79.3%)	0.49
Income <$12 K/year		222 (63.4%)	27 (12.2%)	195 (87.8%)	0.60
HIV Status	Infected	231 (66.0%)	33 (14.3%)	198 (85.7%)	0.62
	Uninfected	119 (34.0%)	14 (11.8%)	105 (88.2%)	
BMI	<30	168 (48.0%)	17 (10.1%)	151 (89.9%)	0.08
	>30	182 (52.0%)	30 (16.5%)	152 (83.5%)	
Marital Status	Yes	100 (20.6%)	11 (11.0%)	89 (89.0%)	0.49
	Other	250 (71.4%)	36 (14.4%)	214 (85.6%)	
Has Household members <18 years old	Yes	139 (39.5%)	24 (17.3%)	115 (82.7%)	0.11
	No	211 (60.3%)	23 (10.9%)	188 (89.1%)	
Stable Housing	Yes	314 (89.7%)	40 (12.7%)	274 (87.3%)	0.30
	No	36 (10.3%)	7 (19.4%)	29 (80.6%)	
Illicit Drug Use	Yes	16 (4.6%)	1 (6.3%)	15 (93.8%)	0.71
	No	334 (95.4%)	46 (13.8%)	288 (86.2%)	
Alcohol Use (at least moderate)	Yes	59 (16.7%)	8 (13.6%)	51 (86.4%)	0.80
	No	291 (83.1%)	39 (13.4%)	252 (86.6%)	
Sex act	Unprotected	117 (33.5%)	12 (10.26%)	105 (89.74%)	0.45
	Protected	94 (26.9%)	15 (15.96%)	79 (84.04%)	
	None	138 (39.5%)	19 (13.77%)	119 (86.23%)	

*Food Secure + Food Insecure without Hunger.

**Including White Hispanic.

***Including Black Hispanic.

Food insecurity was highly prevalent being reported by almost one third of women (110/350, 31%) over the last six months. Over 13% of women reported food insecurity with hunger (Table 1). Women who identified their race as “Other” were more likely to be food insecure with hunger, as compared to white and black women (20.7% vs. 16.1% and 9.9%, respectively, p = 0.04). Of the obese women, 65/182 (36.0%) were food insecure and 30/182 (16.5%) were food insecure with hunger, as compared to non-obese women, of whom 10.1% were food insecure with hunger (p = 0.08, (data not shown)). There were no qualitative or statistical differences between women who were food insecure with (Vs. without) hunger in terms of age, marital status, drug and alcohol use and sexual acts. Moreover, there was little difference in food insecurity with hunger by HIV status, (14.3% vs. 11.8%, p = 0.62 for HIV-infected vs. HIV negative, respectively).

Obesity (BMI≥30) was prevalent in this population of low income, HIV-infected and –uninfected women. Over half were obese with a BMI≥30 (52.0%) (Table 1). In unadjusted analyses, women who had food insecurity with hunger had higher odds of obesity (OR 1.75, 95% CI [0.93, 3.31]) (Table 2). In adjusted analyses, women who were food insecure with hunger had higher odds of obesity (aOR 2.56, 95% CI [1.27, 5.20]) after adjusting for HIV status, age, race, household status, income, drug and alcohol use (Table 2).

**Table 2:** Bivariate and multivariate analysis of factors associated with obesity (BMI≥30 versus <30).

Variable		BMI<30(Baseline)	BMI≥30	p-value	Bivariate		Multivariate	
					OR	95% CI	aOR	95% CI
Age, years		49.7	48.2	0.10	0.90	(0.79, 1.02)	0.84	(0.73, 0.98)
Race/Ethnicity	White**	35 (62.5%)	21 (37.5%)					
	**Black** ***	89 (42.0%)	123 (58.0%)	0.01	2.30	(1.26, 4.22)	**2.49**	**(1.31, 4.74)**
	Other	44 (53.7%)	38 (46.3%)		1.44	(0.72, 2.88)	**1.39**	**(0.67, 2.91)**
**Food insecurity with hunger**		17 (36.2%)	30 (63.8%)	0.09	1.75	(0.93, 3.31)	**2.56**	**(1.27, 5.20)**
Income, >12 K		53 (42.4%)	72 (57.6%)	0.28				
HIV infection		121 (52.4%)	110 (47.6%)	0.02	0.59	(0.38, 0.93)	0.54	(0.33, 0.90)
Household members <18 years		56 (40.3%)	83 (59.7%)	0.02	1.68	(1.09, 2.59)	2.15	(1.02, 4.53)
Stable Housing		147 (46.8%)	167 (53.2%)	0.22	1.59	(0.79, 3.20)		
Drug Use Cocaine, Heroin, Crack		11 (68.8%)	5 (31.3%)	0.12	0.40	(0.14, 1.19)		
Alcohol, moderate		27 (45.8%)	32 (54.2%)	0.92	1.13	(0.64, 2.02)		
Sex act	Unprotected	59 (50.4%)	58 (49.6%)	0.80	0.88	(0.51, 1.52)	0.78	(0.43, 1.43)
	Protected	44 (46.8%)	50 (53.2%)					
	None	64 (46.4%)	74 (53.6%)		1.04	(0.62, 1.76)	1.34	(0.74, 2.42)

**Including White Hispanic.

***Including Black Hispanic.

Black women as compared to White women had higher odds of being obese (aOR 2.49, 95% CI [1.31, 4.74]) (Table 2). HIV infected individuals had lower odds of obesity as compared to HIV negative women (OR 0.54, 95% CI [0.33, 0.90]). Older women also had lower odds of obesity (OR 0.84, 95% CI [0.73, 0.98]). Income, household members under age 18, housing status or drug and alcohol use were not qualitatively or statistically associated with obesity. The Hosmer-Lemeshow goodness of fit test on the final model had p value of 0.54.

## Discussion

Food insecurity with hunger was associated with obesity in this population of HIV infected and HIV-negative urban women.

Obesity has multiple adverse health effects, including increased risk for metabolic syndrome, heart disease, and diabetes, all which also increase with age [29], [30] and can be compounded by the inflammatory affects of HIV itself [31]–[33]. As women with HIV are living longer, they may be disproportionately affected by the combination of food insecurity, obesity and their already increased risk for metabolic syndrome. Our findings are the first that we are aware of to identify the link between food insecurity with hunger and obesity among HIV-infected and at risk women.

The relationship we, and others, observed between food insecurity and obesity may be related to the body’s response to a high metabolic (glucose) load in times of plenty as compared to times of inadequate food. Food insecure households often consume high levels of energy dense foods that are nutritionally lacking, including foods high in saturated fats and refined carbohydrates, increasing the risk for obesity [34]–[35]. Many food insecure households report overeating during times of food availability to compensate for times when food is less available, or they do not have money to buy food [36]. This type of eating, with its related rapid spikes of glucose in the blood and subsequent rapid increases in insulin, may contribute to diet-related disorders such as cardiovascular disease and insulin resistance [37].

Another postulated mediator of obesity among food insecure women is through mental stress. Obesity and psychiatric disorders are thought to have a bi-directional relationship, with a high prevalence of obesity in people with existing mental health disorders, and depression and anxiety contributing to obesity [38]. Multiple studies have shown higher odds of mental stress among food insecure women, including over 3 times higher odds of major depression [39] and higher odds of perceived stress and depressive symptoms [40], [41]. Additionally, people with higher stress levels, often measured as blood cortisol levels, have been shown to increase obesogenic eating habits (eating more high-calorie dense food in times of stress) leading to obesity [42]. In qualitative studies across cultures, food insecurity was associated with themes of anxiety, alienation and shame [43]–[45]. Further studies are needed to understand the relationship between food insecurity, mental health and obesity.

Participation in food assistance programs, such as the Supplemental Nutrition Assistance Program (SNAP) (formerly known as food stamps) helps to alleviate food insecurity [8]. While there are some studies suggest that food assistance programs are a contributing factor to the obesity problem among low income women [46]–[47], many use historical data and do not control for food insecurity [47].

There have also been many analyses showing the opposite. One analysis showed that the mean BMI was in fact lower among patients receiving higher amounts of SNAP benefits ($150/month) [4]. This might indicate that if women are only receiving a small amount of food supplementation benefits per month, they are more likely to eat the high calorie dense rich foods that are often cheaper than fresh food, which could lead to obesity [34]. Another study showed that when food insecurity was controlled for, BMI was lower in adults who were enrolled in SNAP >6 months compared to those enrolled for <6 months [48], implying that adequate SNAP benefits may lower the risk of obesity. Additionally, a study in New York City showed that food insecure women not receiving food assistance had higher BMIs as compared to those who were food insecure and receiving food assistance [49]. The Institute of Medicine has recently released a report summarizing the debate in the literature on SNAP benefits and obesity and coming out in support of food assistance programs to address food insecurity [8].

Our finding that Black women had higher odds of obesity is consistent with national statistics: Black women are 80% more likely to be obese than White women [50]. Food insecurity disproportionally affects Black households, with up to a quarter reporting food insecurity and 10% reporting hunger [1]. Black women are also the subpopulation most affected by HIV/AIDS with Black women accounting for almost 30% of all new infections among Black Americans, 20x the rate of white women [51]. Clearly the health effects of HIV, obesity and food insecurity in Black women needs further investigation.

Interestingly HIV conferred slight protection against obesity in our study, although the prevalence of obesity in HIV infected individuals was quite high at 48%. Other studies have shown that prevalence of obesity in HIV infected individuals is comparable to the general population and more common in African-American women [23], similar to our findings.

This study has several limitations. Due to the cross-sectional design, we could not determine causality. We also did not measure participation in food assistance programs, but as stated above, receiving high levels of SNAP benefits has not been found to be consistently associated with obesity in other studies [4]. This study differs from other studies evaluating food insecurity among HIV-infected populations as this population did not exhibit typical risk factors for food insecurity including drug and alcohol abuse, risky sexual practices, and unstable housing [15], which may limit its generalizability.

Understanding the mechanisms for how food insecurity may contribute to obesity among HIV-infected and uninfected low income women is very important due to the known adverse health effects of obesity. Obesity and its contributions to the pro-inflammatory state of HIV disease may be very important for understanding HIV progression [32]. Food insecurity is an independent marker for increased mortality [52] and the repercussions of being both food insecure and obese need to be further evaluated.
